# The histone methyltransferase Setd8 alters the chromatin landscape and regulates the expression of key transcription factors during erythroid differentiation

**DOI:** 10.1186/s13072-020-00337-9

**Published:** 2020-03-16

**Authors:** Jacquelyn A. Myers, Tyler Couch, Zachary Murphy, Jeffrey Malik, Michael Getman, Laurie A. Steiner

**Affiliations:** 1 Center for Pediatric Biomedical Research, Department of Pediatrics, University of Rochester, Rochester, NY USA; 2 Genomics Resource Center, University of Rochester, Rochester, NY USA

**Keywords:** Erythroid, Setd8, H4K20me1, Chromatin, Differentiation

## Abstract

**Background:**

SETD8 is the sole methyltransferase capable of mono-methylating histone H4, lysine 20. SETD8 and H4K20me1 play a role in a number of essential biologic processes, including cell cycle progression, establishment of higher order chromatin structure, and transcriptional regulation. SETD8 is highly expressed in erythroid cells and erythroid deletion of *Setd8* is embryonic lethal by embryonic day 11.5 (E11.5) due to profound anemia, suggesting that it has an erythroid-specific function. The function of SETD8 in the hemopoietic system is poorly understood. The goal of our study was to gain insights into the function of SETD8 during erythroid differentiation.

**Results:**

We performed ATAC-seq (assay for transposase-accessible chromatin) on sorted populations of E10.5 *Setd8* mutant and control erythroblasts. Accessibility profiles were integrated with expression changes and a mark of heterochromatin (H3K27me3) performed in wild-type E10.5 erythroblasts to further understand the role of SETD8 in erythropoiesis. Data integration identified regions of greater chromatin accessibility in *Setd8* mutant cells that co-located with H3K27me3 in wild-type E10.5 erythroblasts suggesting that these regions, and their associated genes, are repressed during normal erythropoiesis. The majority of these more accessible regions were located in promoters and they frequently co-located with the NFY complex. Pathway analysis of genes identified through data integration revealed stemness-related pathways. Among those genes were multiple transcriptional regulators active in multipotent progenitors, but repressed during erythroid differentiation including *Hhex, Hlx*, and *Gata2*. Consistent with a role for SETD8 in erythroid specification, SETD8 expression is up-regulated upon erythroid commitment, and Setd8 disruption impairs erythroid colony forming ability.

**Conclusion:**

Taken together, our results suggest that SETD8 is an important regulator of the chromatin landscape during erythroid differentiation, particularly at promoters. Our results also identify a novel role for Setd8 in the establishment of appropriate patterns of lineage-restricted gene expression during erythroid differentiation.

## Introduction

Hematopoiesis is the process by which differentiated blood cells are generated from multipotent stem and progenitors. During hematopoiesis, populations of stem and progenitor cells with self-renewal potential become increasingly lineage restricted, eventually giving rise to terminally differentiated progeny (reviewed in [3]). The coordinated action of transcriptional regulators and epigenetic modifiers regulates the path a cell takes as it transcends the hematopoietic hierarchy, with terminal differentiation requiring silencing of the multilineage transcriptome and activation of the transcriptional program governing terminal differentiation. Highlighting this fact, mutations that disrupt epigenetic or transcriptional regulation in multipotent hematopoietic stem and progenitor cells can result in leukemia, myelodysplastic syndromes, and other types of bone marrow failure [4–7].

In eukaryotes, DNA is packaged into chromatin by histone proteins. The core unit of chromatin is the nucleosome, composed of 147 bp of DNA wound around an octamer of two copies of each histone H2A, H2B, H3, and H4. The “tails” of these histone proteins can be post-translationally modified altering the function of the associated DNA. Methylation of histone H3 on lysines 4, 9, and 27 are among the most studied posttranslational modifications, with well-defined consequences on chromatin structure and gene expression. Several mediators of histone H3 methylation, such as the polycomb and MLL complexes, have well-characterized roles in both normal and malignant hematopoiesis [8, 9]. SETD8 is the sole methyltransferase in mammals capable of mono-methylating histone H4, lysine 20 (H4K20me1) [10]. SETD8 and H4K20me1 are involved in a diverse and important array of biologic processes including cell cycle progression, DNA damage repair, higher order chromatin structure, and transcriptional regulation (reviewed in [11, 12]). SETD8 is essential for erythropoiesis. Erythroid deletion of SETD8 is lethal due to severe anemia, with SETD8 null erythroblasts having decreased viability, altered cell cycle progression, and impaired nuclear condensation and maturation [13]. In addition, Setd8 is a known repressor of the master regulator GATA2 during terminal erythroid maturation [14, 15]. In contrast to the large body of work on histone H3 methylation, however, the function of SETD8 and histone H4 methylation during hematopoietic differentiation have been much less explored, and the function of SETD8 during erythroid differentiation remains incompletely understood. In this study, we sought to delineate the function of SETD8 during erythroid commitment and differentiation, and observed an unexpected role in regulating the chromatin landscape and suppressing genes whose expression is typically restricted to other parts of the hematopoietic hierarchy.

## Results

### Setd8 mutant erythroblasts have altered chromatin accessibility

**Fig. 1:**
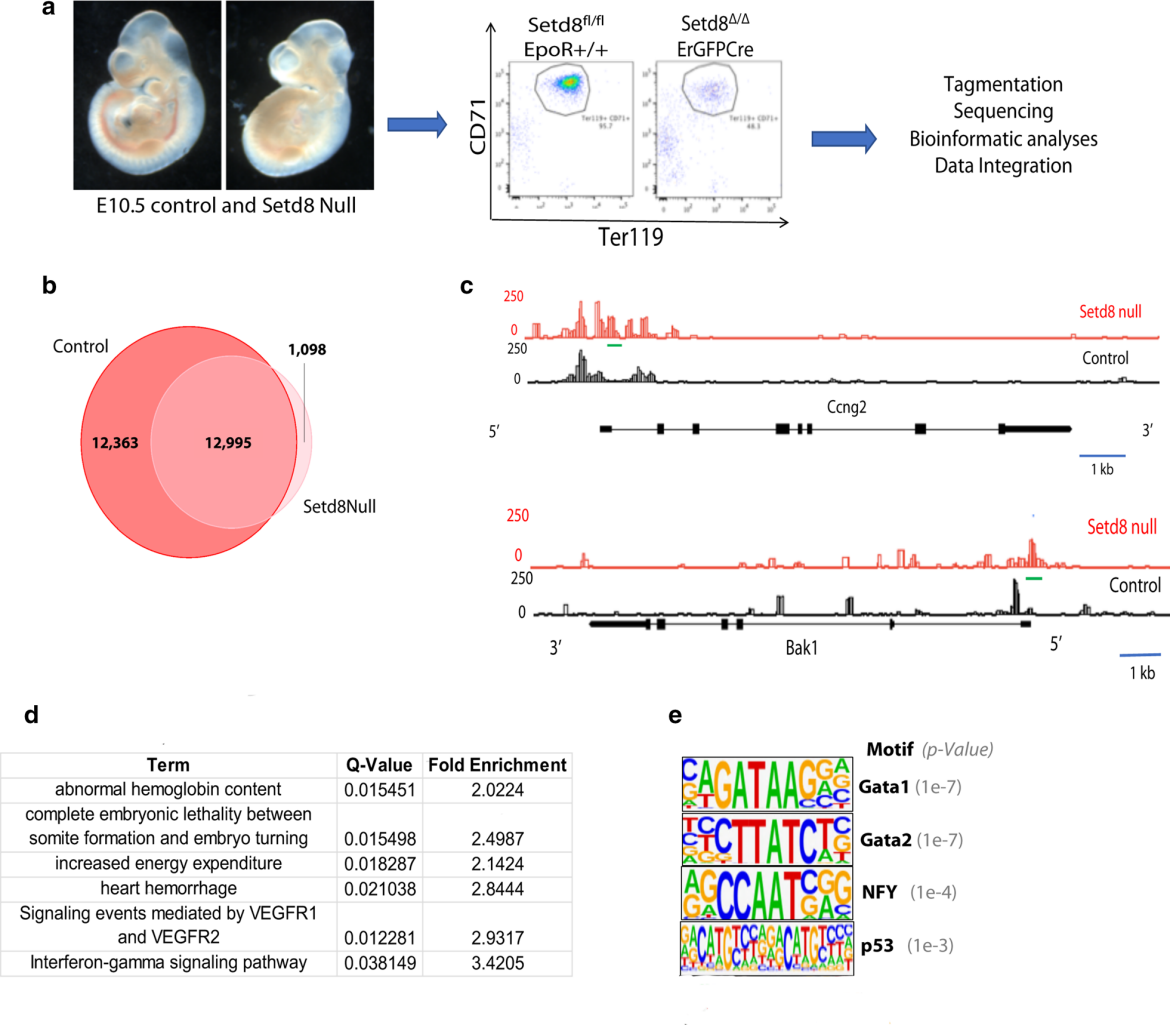
Loss of *Setd8* alters the chromatin landscape in erythroblasts. **a** Experimental design of ATAC-seq experiments. Early erythroblasts were sorted from the blood of *Setd8* mutant and control embryos, and subjected to tagmentation and sequencing as outlined in [1]. **b** Overlap of accessible regions in *Setd8* mutant and control. **c** Chromatin accessibility at the Ccng2 and Bak1 loci. Green line highlights regions of chromatin accessibility present in *Setd8* mutant but not control. **d** Significantly enriched pathways based on regions of accessibility exclusively in *Setd8* mutant erythroblasts. **e** Significantly enriched motifs based on regions of accessibility exclusively in *Setd8* mutant erythroblasts

Erythroid deletion of SETD8, achieved by crossing mice that harbor floxed *Setd8* alleles [10] with mice that that express cre-recombinase under the direction of the endogenous erythropoietin promoter, [16] is embryonic lethal by embryonic day 11.5 (E11.5) due to profound anemia [13]. To gain insights into the function of SETD8 during erythropoiesis, we performed assay for transposase-accessible chromatin using sequencing (ATAC-seq) on erythroblasts sorted from the blood of E10.5 *Setd8* mutant (Setd8 fl/fl: EpoRCre) and control (Setd8 fl/+;EpoRcre, Setd8 fl/fl) embryos (Fig. 1a). Cell number for the *Setd8* mutant samples was limited due to severe anemia, with ~ 1000 cells used for each replicate. *Setd8* mutant and control replicates were aggregated and 14,093 and 25,358 accessible regions identified, respectively. As the littermate control embryos were not anemic, we were able to utilize ~ 25,000 cells per replicate; it is likely that the lower number of accessible regions identified in the *Setd8* mutant cells is secondary to decreased assay sensitivity due to low cell number. Despite the limited number of mutant cells, extensive accessibility overlap was observed, with 92% of regions identified in *Setd8* mutant cells also identified in control (Fig. 1b).

We focused our initial analyses on the 1098 regions only accessible in the *Setd8* mutant erythroblasts. Examples of these regions are shown in Fig. 1c. Consistent with the phenotype of the *Setd8* mutant embryos, significant functional predictions of the 1098 regions include “abnormal hemoglobin content” and “complete embryonic lethality between somite formation and embryo turning” (Fig. 1d). Motif enrichment analysis identified significant enrichments for the transcription factors GATA1 (1e−7) and GATA2 (1e−7), which are both essential for erythropoiesis [17, 18]. In addition, a significant enrichment for the p53 binding motif (1e−3) was identified, consistent with the signature of p53 activation previously observed in the transcriptomic evaluation of the *Setd8* mutant erythroblasts [13]. Taken together, these data suggest that specific cis-regulatory regions accessible only in *Setd8* mutant cells are associated with both normal and dysregulated erythropoiesis.

**Fig. 2:**
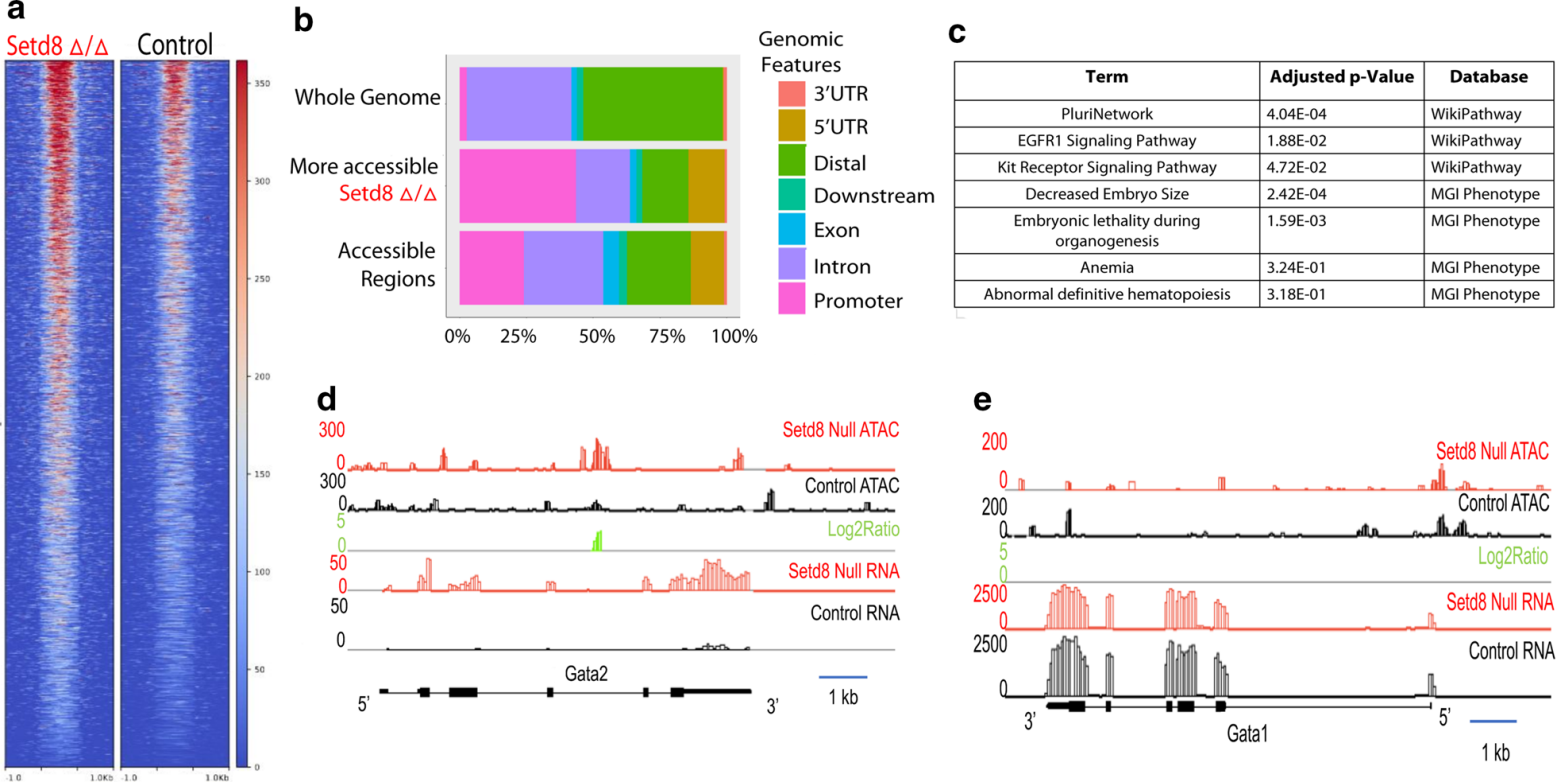
Regions of increased chromatin accessibility in *Setd8* mutant cells compared to controls. **a** Heat map of normalized chromatin accessibility in *Setd8* mutant and control erythroblasts. **b** Genomic distribution of regions more accessible in *Setd8* mutant cells and all accessible regions identified compared to the overall composition of the whole genome. **c** Significantly enriched pathways based on genes co-located with regions of more accessible chromatin. **d**–**e** Chromatin accessibility at the *Gata2* and *Gata1* loci. At the Gata2 locus, there is a region of increased chromatin accessibility in *Setd8* mutant erythroblasts that is associated with increased mRNA expression. In contrast, chromatin accessibility and mRNA expression at the *Gata1* locus is similar in *Setd8* mutant and control samples

To gain further insights into how *Setd8* deletion alters the erythroid chromatin landscape, differentially accessible regions were identified by computing a log2 ratio between mutant and control for all enriched regions. As identification of regions with lower chromatin accessibility in *Setd8* mutant samples was likely to be confounded by decreased assay sensitivity due to low cell number, we focused on regions with more accessibility in *Setd8* mutant compared to control. In total, we identified 4462 regions with more chromatin accessibility in the *Setd8* mutant cells (Fig. 2a), based on log2 sum score for the region of greater or equal to 15. The regions more accessible in the *Setd8* mutant samples were most commonly located at promoters, and less commonly found in introns and distal intergenic regions (Fig. 2b). Pathway and ontology enrichment analysis of genes nearby more accessible mutant regions were significant for erythropoiesis related terms, including “anemia” and “definitive hematopoiesis” as well as development and differentiation related pathways including “plurinetwork”, “decreased embryo size”, and “complete embryonic lethality during organogenesis” (Fig. 2c). Despite increased chromatin accessibility and mRNA expression at loci that are typically repressed during erythroid specification, such as *Gata2* [19] (Fig. 2d), chromatin accessibility and RNA expression was similar in mutant and control samples at several key erythroid loci, such as Gata1 (Fig. 2e), suggesting that the observed changes in the chromatin landscape were not due solely to delayed maturation.

### Chromatin accessibility is altered at differentially expressed genes

**Fig. 3:**
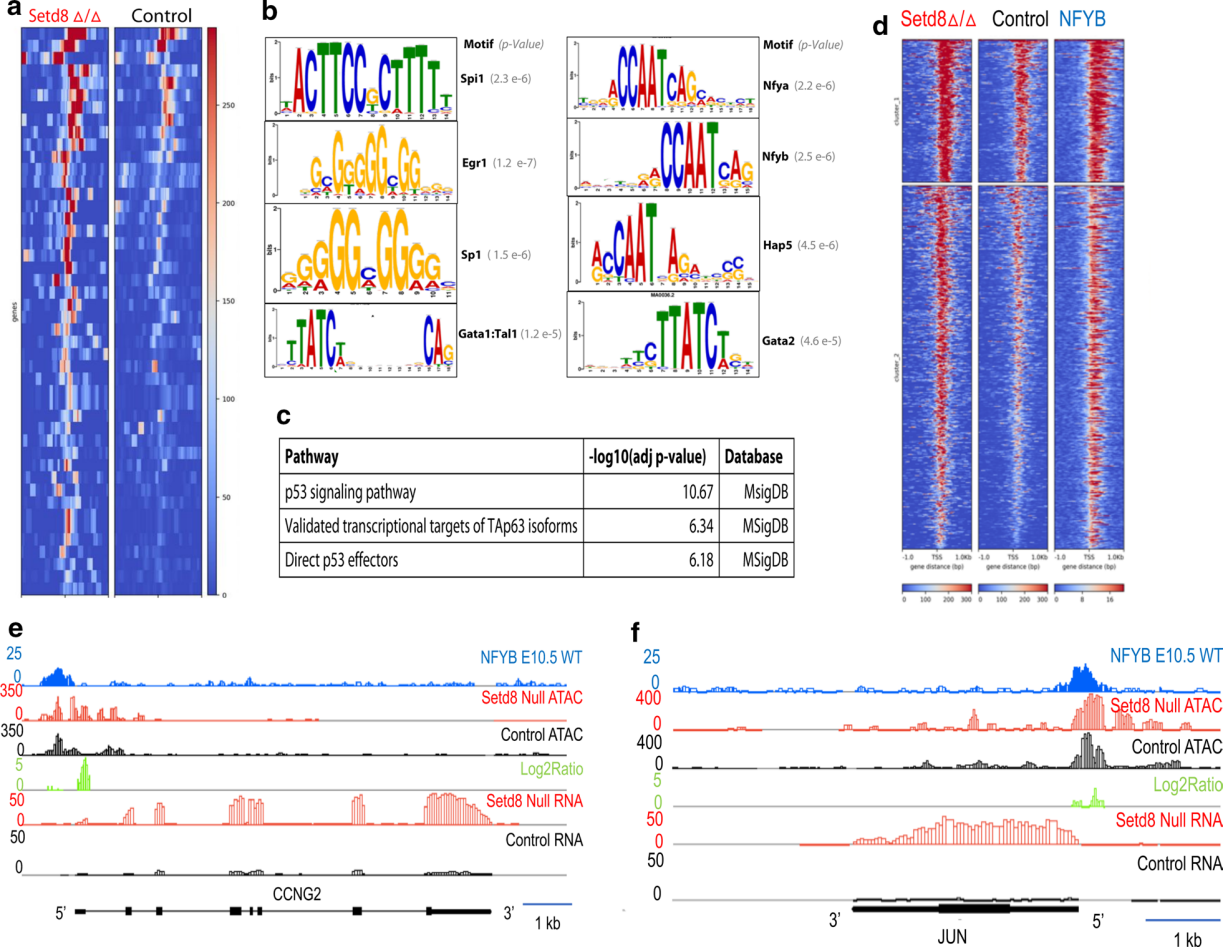
Candidate cis-regulatory elements responsible for gene expression changes. **a** Heat map of normalized coverage based on chromatin accessibility at differentially expressed genes. **b** Enriched DNA motifs at regions of differentially accessible chromatin that co-locate with differentially expressed genes. **c** Significantly enriched pathways and motifs based on regions of increased accessibility that are co-located with differentially expressed genes. **d** Heat map of normalized coverage illustrates co-location of NFYB with regions of increased accessibility in *Setd8* mutant erythroblasts. **e**, **f** Chromatin accessibility at the Ccng2 and Jun loci demonstrating a peak of increased chromatin accessibility co-located with a region of NFYB occupancy

To further delineate the relationship between the chromatin landscape and gene expression, we compared regions of increased accessibility with genes differentially expressed between mutant and control, [13] identifying 361 regions of increased chromatin accessibility that overlapped with a differentially expressed gene (Fig. 3a; Additional file 1: Table S1). Among these genes were 30 known GATA2 target genes (Additional file 2: Table S2), consistent with the established role of SETD8 as a *Gata2* repressor [14, 15]. The transcription factors predicted to regulate differentially expressed genes co-located with a more accessible region included GATA2, P53, and the NFY complex (NFYA and NFYB) (Additional file 3: Table S3). Several of these factors were also identified through DNA motif enrichment analyses (Fig. 3b). *Setd8* mutant erythroblasts had evidence of p53 activation including cell cycle abnormalities, increased rates of apoptosis, and accumulation of DNA damage, as well as transcriptomic evidence of p53 activation [13]. Consistent with the phenotype of the *Setd8* mutant cells, “p53 signaling” was the pathway most enriched in genes identified through genomic data integration (Fig. 3c). Intriguingly, “validated transcriptional isoforms of Tap63” was also identified as an enriched pathway (Fig. 3c). p63 is an established regulator of epidermal differentiation whose expression is regulated by Setd8 [20]. Furthermore, ChIPmentation for NFYB supported these data, revealing overlap of NYB enrichments with regions of chromatin more accessible in *Setd8* mutant erythroblasts (Fig. 3d–f). Overall, these data propose candidate cis-regulatory factors that might be responsible for expression changes due to the loss of Setd8 in erythroblasts.

### A subset of regions with increased chromatin accessibly in Setd8 mutant cells co-locate with H3K27me3 in wild-type E10.5 erythroblasts

**Fig. 4:**
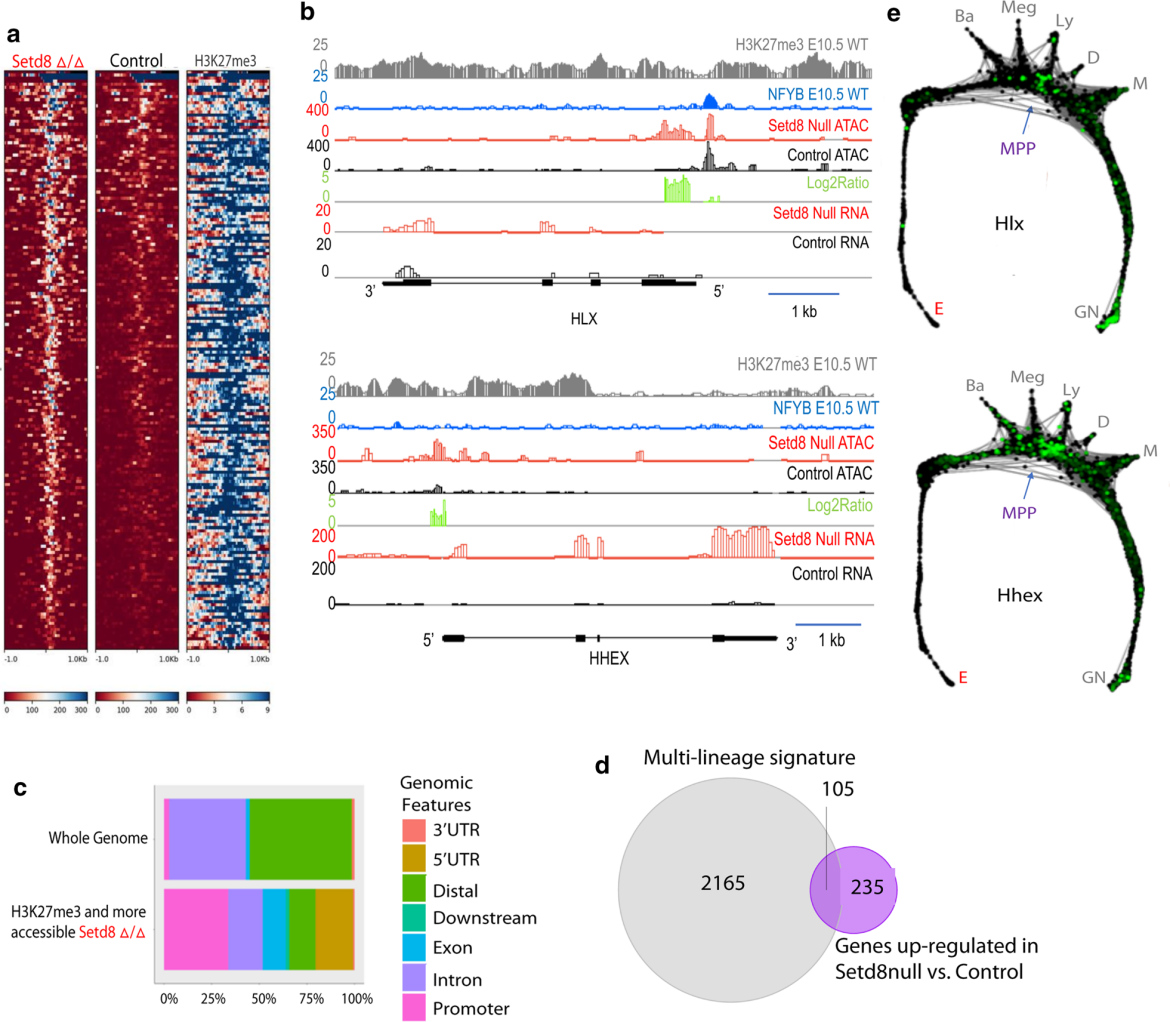
A subset of regions with increased chromatin accessibility in *Setd8* mutant cells are marked by the repressive heterochromatin mark H3K27me3 in wild-type erythroblasts. **a** Heat map of normalized coverage depicting H3K27me3 occupancy at regions of more accessible chromatin in *Setd8* mutant erythroblasts. **b** Chromatin accessibility and H3K27me3 occupancy at the *Hlx* and *Hhex* loci. **c** Genomic distribution of regions more accessible in *Setd8* mutant that contain significant enrichments of H3K27me3 in normal erythroblasts. Results are compared to the composition of the whole genome at baseline. **d** Overlap of genes expressed at higher levels in multipotent progenitors compared to proerythroblasts with genes expressed more highly in *Setd8* mutant cells compared to control. **e** scRNA-Seq performed on Kit+ hematopoietic progenitors from the mouse bone marrow confirms that candidate genes (*Hhex* and *Hlx*) are members of the multilineage transcriptome that are repressed during erythroid specification. Brighter green color indicates increased RNA expression. Data were adapted from: https://kleintools.hms.harvard.edu/paper_websites/tusi_et_al/; [2])

To further determine the impact of Setd8 disruption of the chromatin landscape, ChIPmentation [21] was used to identify regions of H3K27me3 occupancy, a marker of repressed heterochromatin, in wild type (WT) E10.5 erythroblasts. Integration of these data with chromatin accessibility identified several regions that are more accessible in *Setd8* mutant cells and exist as heterochromatin in WT E10.5 erythroblasts, suggesting that these regions should be repressed during normal erythropoiesis (Fig. 4a). Upon further evaluation, several of these regions co-localized with genes that are up-regulated in the *Setd8* mutant cells including *Hhex CD63*, and *Gata2*. In addition, we identified several transcriptional regulators active in multipotent hematopoietic progenitors but typically silenced during erythroid differentiation including *Cebpa* and *Hlx* (Fig. 4b and Additional file 4: Fig. S1). Regions of increased chromatin accessibility in mutant cells that co-located with H3K27me3 in wild-type erythroblasts were located primarily at the promoter or 5′ UTR (Fig. 4c). Pathway enrichment analysis based on genes living within ± 10 kb of regions that are more accessible in *Setd8* mutant and contain enrichments for H3K27me3 in wild-type erythroblasts identified several stemness-related pathways including “Transcriptional regulation of pluripotent stem cells” and “OCT4, SOX2, NANOG repress genes related to differentiation” (adjusted *p* 0.005 and 0.008, respectively). Taken together, these data suggest that Setd8 participates in the establishment of appropriate patterns of lineage-restricted gene expression during erythroid differentiation.

### Setd8 mutant erythroblasts do not effectively silence the multilineage transcriptome

Given the findings that regions of increased accessibility in *Setd8* mutant erythroblasts are significantly enriched for stemness-related transcription factors and pathways, we next compared the differentially expressed genes in *Setd8* mutant erythroblasts to transcriptomic changes that occur as a cell transcends the hematopoietic hierarchy, gaining lineage specificity while suppressing the multilineage transcriptome. Global transcriptomic data on emerging hematopoietic progenitors in the early embryo are extremely limited. As a proxy, a gene signature was identified that contained genes up-regulated in the multipotent bone marrow progenitors compared to proerythroblasts (GSE14833). Consistent with the *Setd8* mutant altered chromatin landscape, a large fraction, 105/340 (~ 30%), of the differentially expressed genes in the *Setd8* mutant erythroblasts, are up-regulated in the previously defined multilineage transcriptome (Fig. 4d). Furthermore, due to the advent of single cell RNA-Seq technologies, (https://kleintools.hms.harvard.edu/paper_websites/tusi_et_al/; [2]) expression profiles evaluated at the single cell resolution supported that these genes are typically down-regulated upon erythroid lineage commitment (Fig. 4e).

Cell-type enrichment analyses performed on genes robustly expressed across all samples (1835 genes with CPM>6) identified megakaryocyte erythroid progenitor (MEP; Additional file 4: Fig. S2A) as the most significantly enriched cell-type. Of note, the Enrichr platform used to determine cell-type enrichment does not contain erythroblast data; the most closely related cell-type in Enrichr is the MEP. In contrast, cell-type enrichment analyses on the differentially expressed genes in the *Setd8* mutant erythroblasts (288 genes with CPM>6) was significant for other myeloid cell types derived from the upstream multipotent common myeloid progenitor (CMP), most notably mast cells, as well as other non-erythroid cells (Additional file 4: Figure S2B). These data further suggest that *Setd8* mutant erythroblasts do not effectively silence the multilineage transcriptome.

### Setd8 disruption in CD34+ hematopoietic stem and progenitor cells impairs erythroid commitment

**Fig. 5:**
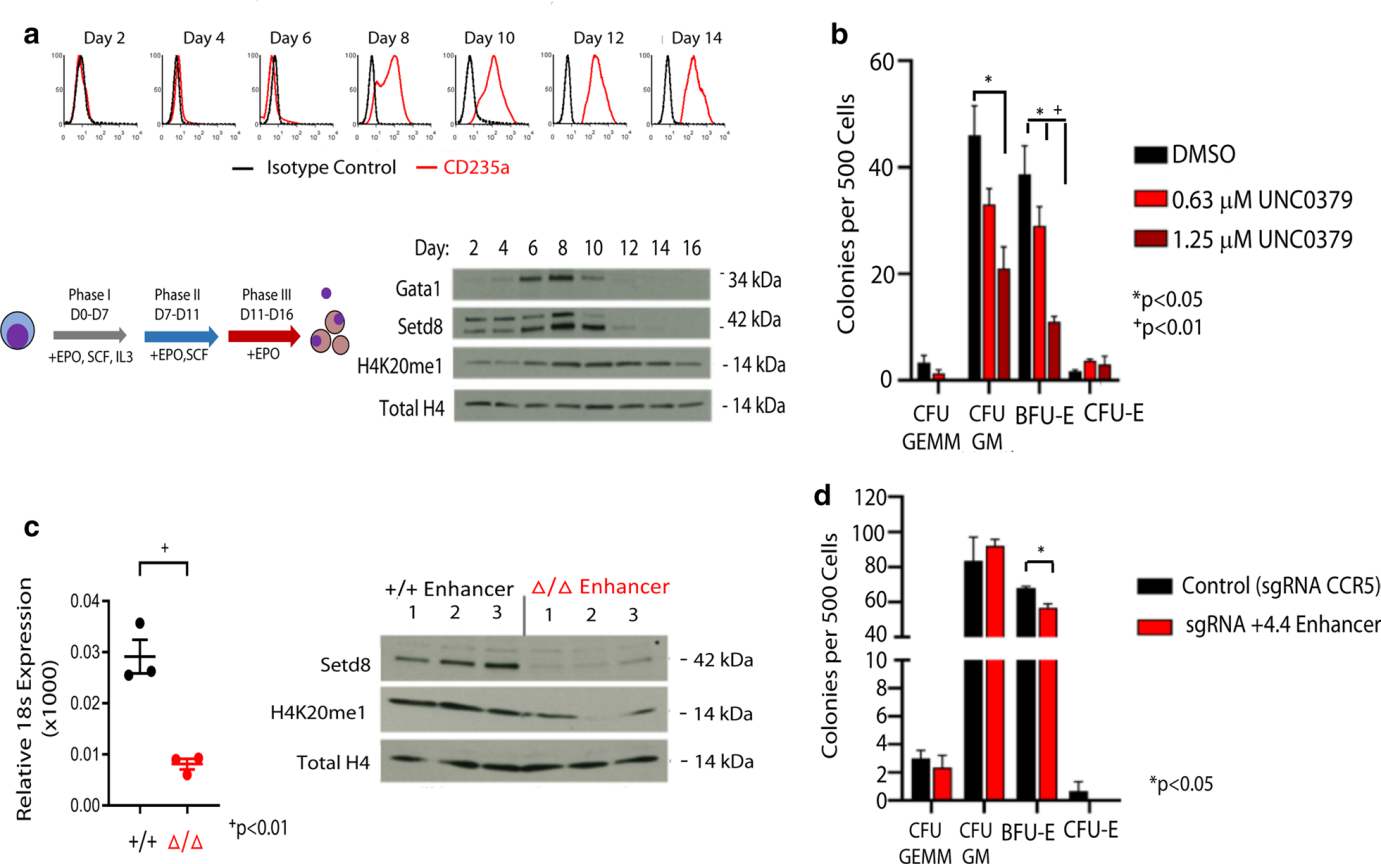
Erythroid differentiation is associated with high-level Setd8 expression. **a** Levels of SETD8, GATA1, and H4K20me1 during erythroid differentiation of CD34+ HSPCs. Cultures were serially monitored by flow cytometric analyses for Glycophorin A (GYPA) expression and the levels of SETD8, GATA1, and H4K20me1 determined by western blotting. Equivalent protein content was loaded in each lane and total H4 was used as a loading control. **b** Colony forming assays following treatment of CD34+ HSPCs with the Setd8 inhibitor UNC0379. *n* = 3 replicates. Error bars represent standard error of the mean (SEM). Significance determined via students *T* test. **c** Deletion of the *Setd8* + 4.4 enhancer results in decreased mRNA (left) and protein expression (right), as well as reduced levels of H4K20me1. *Indicates suspected non-specific cross-reacting band. Three independent enhancer deletion clones were analyzed. Error bars represent SEM. Significance determined via students *T* test. **d** Colony forming assays following genome editing for the SETD8 erythroid enhancer in CD34+ HSPCs. *N* = 3 replicates. Error bars represent SEM. Significance determined via students *T* test

Given our data implicating Setd8 in the repression of the multilineage transcriptome, we next sought to determine the expression pattern of Setd8 during erythroid commitment and differentiation. Interrogation of multiple data sets (GSE14833, GSE14833, GSE6506, GSE14833 [22]) demonstrates that Setd8 mRNA expression increases in abundance as cells undergo differentiation to erythroblasts from multipotent progenitor cells (Additional file 4: Fig. S3A–C). To delineate changes in Setd8 protein levels during erythroid specification, we utilized a well-characterized culture system where erythroblasts are derived from CD34+ hematopoietic stem and progenitor cells (HSPCs) [23]. In this culture system, Setd8 protein expression increases during erythroid commitment, in concert with GATA1 levels and the onset of cell surface expression of Glycophorin A. A corresponding increase in H4K20me1 follows the increase in Setd8 expression, and persists throughout the erythroid maturation phase of the culture (Fig. 5a).

To determine if Setd8 function is necessary for erythroid commitment, we disrupted Setd8 in human CD34+ HSPCs and then assessed erythroid colony forming ability. We first disrupted Setd8 using inhibitor treatment, by plating CD34+ HSPCs in methylcellulose with cytokines capable of supporting multilineage specification and either DMSO or the Setd8 inhibitor UNC0379 (Additional file 4: Fig. S4) [24, 25]. Setd8 inhibition decreased erythroid colony formation in a dose-dependent manner (Fig. 5b). With inhibitor treatment, however, the total number of non-erythroid colonies was also decreased, raising the possibility of off target effects, or global toxicity from Setd8 inhibition. Deletion of the methyltransferase domain using genome editing [26] appeared to have a more specific effect on erythroid colony forming ability (Additional file 4: Figure S5).

We next sought to decrease Setd8 expression in a more targeted manner, by disrupting the enhancer that drives high-level Setd8 expression. To that end we identified a GATA1-TAL1 peak located at + 4.4 kb from the Setd8 promoter in cultured human erythroblasts (Additional file 4: Fig. S6A). This region was confirmed to have enhancer activity in a luciferase assay that was dependent on the GATA1-TAL1 motif (Additional file 4: Fig. S6B). Deletion of this region in the HUDEP2 cell line using genome editing [26] resulted in a significant decrease in the expression of Setd8 and the level of H4K20me1 (Additional file 4: Figs. S7A–D and 5C). We then targeted the + 4.4 Setd8 enhancer using genome editing in CD34+ HSPCs. Following 24 h of recovery, those cells were plated in methylcellulose with cytokines capable of supporting erythroid specification. CD34+ cells where the CCR5 “safe harbor” locus was targeted were used as controls, as homozygous deletion of this region has no obvious hematologic phenotype [27, 28]. Disruption of the erythroid Setd8 enhancer leads to a significant decrease in the efficiency of erythroid colony formation (Fig. 5d and Additional file 4: Fig. S8), without impacting the efficiency of non-erythroid colony formation, supporting a role for Setd8 in erythroid specification.

## Discussion

In this report, we demonstrate that disruption of Setd8 is associated with regions of increased chromatin accessibility that are most commonly located at promoters. These regions frequently co-locate with H3K27me3 enrichment in wild-type erythroblasts, suggesting these regions, and their associated genes, should be repressed during normal erythroid differentiation. Consistent with these observations, Setd8 mutant erythroblasts express genes, including several key transcriptional regulators, that are typically silenced during erythroid lineage commitment. We further demonstrate that Setd8 expression is up-regulated upon erythroid commitment and that disrupting Setd8 in CD34+ HSPCs impairs erythroid colony forming ability. Taken together, these data point to a novel role for Setd8 in regulating the expression of the multilineage transcriptome during erythroid commitment and differentiation.

SETD8 and H4K20me1 have been implicated in the regulation of cellular differentiation in other contexts, including epidermal stem cell differentiation and adipogenesis [20, 29]. Similar to erythroid progenitors, deletion of *Setd8* in murine epidermis results in p53 activation and impaired differentiation [20.] SETD8 can also methylate non-histone targets, most notably, Setd8 methylates p53 at lysine 382. Disruption of this methylation results in p53 activation, [30] and may underlie the common finding of p53 activation in Setd8 mutant erythroblasts and epidermis. Of note, *Setd8* mutant erythroblasts had changes in both the transcriptome and chromatin landscape consistent with p53 activation, however p53 deletion is not sufficient to rescue *Setd8* mutant erythroblasts [13], suggesting that SETD8 regulates other essential processes during erythropoiesis. SETD8 has also been implicated in adipocyte differentiation. Interestingly, the transcription factor GATA2 is a key transcriptional regulator both in early erythropoiesis and in adipogenesis [31]. *Gata2* is repressed by SETD8 during erythroid maturation [14, 15], however disruption of Setd8 expression via siRNA in 3T3-L1 cells, an in vitro model of adipogenesis, does not change *Gata2* expression, [29] highlighting the context dependent nature of SETD8 function. To date, much of the work exploring the function of SETD8 has been conducted in immortalized cell lines, which frequently have karyotypic and other abnormalities, and are often limited in their capacity for terminal differentiation. The ability to obtain populations of stem and progenitor cells with well-defined immunophenotypes makes the hematopoietic system an excellent system to study the consequences of SETD8 disruption outside of the context of immortalization.

To gain insights into the function of SETD8 in erythroid differentiation, we identified regions of increased chromatin accessibly in the *Setd8* mutant cells by comparing a log2 ratio between aggregated *Setd8* mutant and control samples. Increased chromatin accessibility manifested as both narrow peaks with higher read counts and peaks where the enrichments were broader when compared to control. These changes likely represent regions where the population of cells is more homogenous (i.e., the majority of cells at a given locus have open chromatin) or regions occupied by different DNA binding proteins in *Setd8* mutant and control cells. Intriguingly, the majority of these more accessible regions were located in promoters and many of those regions were co-located with the NFY complex. The NFY complex is a trimeric transcription factor that has a histone-like conformation and is composed of NFYA, NFYB, and NFYC. All three members of the complex are required for NFY to bind target promoters, which contain the “CCAAT” motif (reviewed in [32]). NFY is an essential regulator of hematopoietic stem cell proliferation and survival [33] that also has critical roles in more differentiated cells, such as erythroblasts where it is an important regulator of gamma globin expression [34, 35]. Similar to *Setd8*, RNA expression of the NFY subunits increases during erythroid differentiation (bloodspot.eu, data sets GSE14833 and GSE6506). NFY also regulates the expression of several cell cycle genes, and directly interacts with p53 [36]. The similar expression patterns of SETD8 and NFY, their participation in similar cellular pathways, and the frequent co-location of NFY with the more accessible regions of chromatin in Setd8 mutant cells make it tempting to speculate that SETD8 and NFY function cooperatively during erythroid differentiation.

The mechanisms by which SETD8 and H4K20me1 regulate gene expression are incompletely understood. Following mono-methylation by SETD8, H4K20 can be progressively methylated to H4K20me3 by the enzymes SUV420H1 and SUV420H2 [37]. H4K20me3 is generally associated with transcriptional repression and heterochromatin. The role of H4K20me1 in transcriptional regulation appears to be more complex, and through interaction with various reader proteins such as L3MBTL1 and hMOF, H4K20me1 can contribute to both transcriptional repression and activation [11, 12, 38, 39]. In this work, we focused primarily on the role of SETD8 in transcriptional repression due to the dramatic upregulation of genes observed in the *Setd8* mutant cells compared to the controls [13]. Many of these genes contained regions of differentially accessible chromatin, however as SETD8 preferentially binds nucleosomes and has no known DNA binding motif, it is difficult to distinguish direct SETD8 targets from indirect effects, such as the failure to repress *Gata2*. Unfortunately, to date, reliable ChIP-seq data for SETD8 has been elusive. It is also important to note that SETD8 can also interact with non-histone targets, including p53 [30], androgen receptor, [40] PRDM2, [41] and PCNA [42]. Disruption of Setd8’s interaction with these proteins or other non-histone binding partners may be contributing the abnormal chromatin landscape of the Setd8 mutant erythroblasts. Future work to determine SETD8 interactors in erythroid cells, the relationship of SETD8 occupancy to gene expression pattern, and the relationship of SETD8 with key hematopoietic regulators is likely to provide important insights into the epigenetic mechanisms that govern erythroid differentiation.

## Conclusions

Our data demonstrate that SETD8 is an important regulator of the chromatin landscape during erythroid differentiation and that Setd8 mutant cells fail to effectively repress the multilineage transcriptome. Together, these data identify a novel role for Setd8 in the establishment of appropriate patterns of lineage-restricted gene expression during erythroid differentiation. These data provide important insights into the role of Setd8 in regulating local chromatin structure and gene expression as well as novel insights into the molecular mechanisms that regulate erythroid differentiation.

## Methods

### Generation of timed embryos, embryo dissection, and cell sorting

The University of Rochester’s Committee on Animal Resources approved all experiments utilizing mice. To conditionally delete Setd8 in erythroblasts, mice harboring floxed alleles of *Setd8* (Setd8 fl/fl [10]) were crossed with mice that express cre-recombinase under the direction of the endogenous erythropoietin receptor promoter (EpoRCre; [16]). EpoRCre; *Setd8* fl/+ and *Setd8* fl/fl mice were bred overnight and vaginal plugs checked after 12 h (Embryonic Day 0.5; E0.5). At E10.5, the pregnant dam was anesthetized and killed via cervical dislocation and the embryos dissected for further analyses. Following removal of decidual tissues and the placenta in PB2 [Dulbecco PBS (Gibco-BRL), 0.3% BSA (Gemini Bio-Products), 0.68 mM CaCl2 (Sigma-Aldrich), 0.1% glucose, and 12.5 μg/mL of heparin], fetal blood was collected for analyses as it bled from the umbilical and vitelline vessels, as described [43]. Genotyping was performed as previously published [13]. Cells were stained with anti-CD71 (eBiosciences), anti-Ter119 (BD Biosciences), and Dapi (Molecular Probes) and collected via fluorescent cell sorting on a FACSAria II (BD Biosciences).

### Library construction: ATAC-seq and ChIPmentation

Libraries for ATAC-seq were prepared as outlined in [1]. Briefly, nuclei were collected from *Setd8* mutant and control erythroblast following cell lysis and subjected to tagmentation using transposase (Illumina Nextera FC121–1030). Samples were then amplified × 5 PCR cycles [98 ℃/45 s + 5 × (98 ℃/15 s + 63 ℃/30 s + 72 ℃/30 s) + 72 ℃/1 min] using KAPA HiFi HotStart ReadyMix PCR Kit (KK2601/KK2602) and cleaned with Qiagen MiniElute column. The library was then amplified with PCR [98 ℃/45 s + ***Y*** × (98 ℃/15 s + 63 ℃/30 s + 72 ℃/30 s) + 72 ℃/1 min], with the number of cycles (*Y*) determined through via a qPCR library amplification test. The resulting PCR product was cleaned using Agencourt AMPure XP magnetic beads (Beckman Coulter A63880) and subjected to Bioanalyzer analyses prior to sequencing. For ChIPmentation, libraries were prepared as outlined in [21]. Briefly, chromatin immunoprecipitation was done using 1–5 × 10^5^ cells, as outlined in [44]. Following immunoprecipitation using H3K27me3 (Cell Signaling Technology, 9733S) or NFYB antibody (Diagenode, C1540241), tagmentation was done using transposase (Nextera, Illumina), followed by elution from magnetic beads. Samples were then decrosslinked using proteinase K for 2 h at 55 ℃ and the DNA purified using the Qiagen minielute kit. Following clean up, they were subjected to the ATAC protocol, as outlined above.

### Sequencing, alignment, and other bioinformatic analyses

Sequencing was performed using the HiSeq2500v2 and reads were converted to the fastq format using bcl2fastq. Raw reads were processed using trimmomatic (SLIDINGWINDOW:4:20 TRAILING:13 LEADING:13 ILLUMINACLIP: trimmomatic_adapters.fasta:2:30:10 MINLEN:15) to remove adapters and low-quality bases. Quality reads were aligned to the mm9 reference genome using bowtie1 (-m 1). For ATAC-Seq, all reads aligning to the mitochondrial genome (ChrM) and blacklist regions (defined by [1]) were removed using samtools. All replicate bam files were merged using samtools merge and accessible regions were defined using MACS2 [45] (--nomodel --shift -100 --extsize 200 -f BAM -B -g mm). Samples were normalized based on sequencing depth and a continuous log2 ratio was computed genome wide between setd8 null and control using bamCompare (deeptools). The log2 ratio scores were intersected and summed with the 14,093 accessible regions identified in setd8 null using bedtools map (-o sum). Therefore each of the 14,093 regions had a log2 ratio sum score that could be used for ranking; the higher the sum score the more accessible that region is in setd8 null compared to control. *Setd8* mutant regions were then filtered to identify regions more accessible in setd8 null compared to control based on log2 sum score ≥ 15. For ChIPmentation, 100 k and 500 k samples were aggregated and enrichments were called using MACS2 (-t -c --broad --broad-cutoff 0.1 -n -f BAM -B --g mm). To identify nearby genes, accessible or H3K27me3 enrichments were extended ± 10 kb and bedtools intersect was applied using the RefSeq mm9 annotation.

### Enrichment analysis

Enrichr was used to perform enrichment analysis when a gene list was present (all RNA-Seq related analyses or if nearby genes were defined) [46, 47]. GREAT was used to perform pathway and ontology enrichment analysis when the input was in the BED file format (e.g., 1098 accessible regions only identified in *Setd8* mutant) [48]. Homer [49] (mm9) and meme-ChIP [50] were used for motif enrichment analyses. Distribution of genome wide features was determined using CEAS [51].

### Publicly available data integration

Analyses in this paper used datasets from the following sources: GSE14833, Bloodspot.eu (GSE14833, GSE6506, GSE14833) and https://kleintools.hms.harvard.edu/paper_websites/tusi_et_al/.

### Human HSPC culture

Mobilized CD34-positive cells (attained from Yale Cooperative Center of Excellence in Hematology) were expanded in H3000 (Stem Cell Technologies) supplemented with 1% CC100 containing human recombinant Flt3L, SCF, IL-3, and IL-6 (Stem Cell Technologies) for 4 days. Cells were then cultured as previously described [23]. In brief, base medium composed of IMDM (Invitrogen), AB serum (3%), PB plasma (2%), insulin (10 μg/mL), heparin (3 U/mL), and holo-transferrin (200 μg/mL) was used. Culture days 0 to 7 used base medium supplemented with EPO (3 U/mL), SCF (10 ng/mL), and IL-3 (1 ng/mL), while IL-3 was omitted for culture days 7 to 11. Base medium plus EPO only was used for the remainder of the culture, in addition to increased concentration of holo-transferrin (1 mg/mL).

### Western blot

Protein was isolated with RIPA and protein lysates boiled with Laemmli loading dye (5% 2-ME, Bio-Rad) prior to gel electrophoresis on precast gradient gels (4–15%, Bio-Rad). Protein was transferred onto nitrocellulose membranes (Hybond-ECL, GE Healthcare). Blots were blocked (5% BSA in PBS-T) and washed (PBS-T) in between antibody incubations. Antibodies used were targeting GATA1 (Abcam, ab11852), SETD8 (Cell Signaling Technology, CST-2996S), H4K20me1 (Abcam, ab9051), and total H4 (Abcam, ab70701). A secondary antibody conjugated to HRP (goat anti-rabbit, Bio-Rad) was used to react with HRP chemiluminescent substrate (Novex ECL, Invitrogen) and exposed to film for imaging.

### Luciferase assays

Putative enhancer regions were cloned into the pGL2-promoter vector in both orientations at the KpnI site. Sequence verified constructs were transfected into K-562 cells using Lipofectamine 2000 (Invitrogen). 48 h post-transfection, luciferase was measured using the Dual-Glo^®^ Luciferase Assay (Promega) according to the manufacturer’s instructions.

### RNA isolation and reverse transcription quantitative-PCR

RNA was isolated with the RNeasy kit (Qiagen) following the manufacturer’s protocol with on-column DNase treatment being performed. RT-qPCR was performed using the Luna^®^ Universal One-Step kit (New England Biolabs), using the following primers:

**Table:** 

SETD8 expression Fwd	RT-qPCR	ACAAATGCTCTGGAATGCGTT
SETD8 expression Rev	RT-qPCR	CCGGCTAATGGTTTCCCCTG
18S Fwd	RTq-PCR normalization	TTGACGGAAGGGCACCACCAG
18S Rev	RTq-PCR normalization	GCACCACCACCCACGGAATCG

### HUDEP2 culture and genome editing

HUDEP2 cells [52] and clonal populations were generated and maintained as previously described [53]. In brief, sgRNAs targeting flanking regions of the + 4.4 erythroid-specific *SETDB* enhancer were precomplexed with Cas9 (New England Biolabs Engen Cas9 NLS) and introduce via electroporation into HUDEP2 cells. Monoclonal lines were screened for presence or absence of + 4.4 enhancer [9].

### Colony forming assays

CD34+ cells were thawed into H3000 (Stem Cell Technologies) supplemented with 1% CC100 containing human recombinant FLT3L, SCF, IL-3, and IL-6 (Stem Cell Technologies). 24 h later 150,000 CD34+ HSPC cells were electroporated with precomplexed 20 μM Cas9 (NEB Engen Cas9 NLS) and 50 μM sgRNAs. Genome editing confirmed 24 h later by PCR. 48 h after electroporation bulk cells were plated in MethoCult H4434 containing SCF, IL-3, EPO, and GM-CSF (Stem Cell Technologies). Either DMSO or the SETD8 inhibitor UNC0379 was added to the MethoCult, as indicated. Colonies were counted 14 days later.

## Supplementary information

**Additional file 1.** Differentially expressed genes that contain a region of differential chromatin accessibility.

**Additional file 2: Table S2.** Gata2 target genes that are upregulated in setd8-mutant and have open chromatin higher in setd8-mutant.

**Additional file 3.** Transcription factors predicted to regulate genes that contain a region of differential chromatin accessibility.

**Additional file 4.** Additional figures.

## Data Availability

The data reported in this paper have been deposited in GEO Accession number GSE138106.
