# Supplementary material for: The histone methyltransferase Setd8 alters the chromatin landscape and regulates the expression of key transcription factors during erythroid differentiation
Source: Epigenetics Chromatin. 2020 Mar 16;13:16. doi: 10.1186/s13072-020-00337-9 (PMC7075014; doi:10.1186/s13072-020-00337-9)
Supplement: Supplementary file 1 — Additional file 1. Differentially expressed genes that contain a region of differential chromatin accessibility. [file 13072_2020_337_MOESM1_ESM.pdf]

Rdh10
Dst
Chst10
Nck2
Zranb3
Stx6
Uck2
Uap1
1700034H15Rik
Tmem70
Ptp4a1
Idh1
Rpl37a
Tmbim1
Dnpep
Fbxo36
Itm2c
Dbi
Pm20d1
Mdm4
Btg2
Ube2t
Kif21b
Npl
Rnasel
2810025M15Rik
BC026585
Slc19a2
1110051M20Rik
Phyh
Ass1
B230118H07Rik
Cd59a
Atrn
Smox
Dnmt3b
Dlgap4
Atp9a
Msrb2
BC029214
Egfl7
D330023K18Rik
Bbs5
Ssfa2
Smtnl1
Slc39a13
Creb3l1
Prrg4
Tmem62
Lcmt2

Serf2
Casc4
Sord
Fahd2a
Adam33
Prnp
Dstn
Zfp341
Pxmp4
Romo1
Sys1
Tmem189
Rps21
Rgs19
Rpl22l1
Wwtr1
Etfdh
Trim2
Fhdc1
Vangl1
Vav3
Tpd52
Zfand1
Rab33b
Mfsd1
Lmna
Krtcap2
Pbxip1
Zbtb7b
Shc1
Rab13
Scnm1
Hsd3b6
Dnajb4
Wdr78
B4galt1
Tek
Ptpru
Pafah2
Rps20
Chchd7
Trp53inp1
Pnrc1
Chmp5
Dctn3
Hint2
Gne
Mtap
Tmem53
Iqcc

1700003M07Rik
Gmeb1
Fam76a
Fuca1
Pink1
Tmem51
Acot7
Mib2
Agrn
Klhl7
Bend4
Srd5a3
Ccng2
Pigg
Atp5k
Ulk1
Hscb
Mvk
Rab35
Acad12
Gpn3
Fis1
Lmtk2
Rasl11a
St7
Cald1
Tspan12
Rtkn
Mkrn1
Tigd2
Abtb1
Zfp9
Ptpn6
Ccnd2
Arhgdib
Ldhb
Gpi1
Gys1
Lrrc28
Eed
Fam168a
Ndufab1
Rps9
Leng1
Tmc4
Ube2s
Zfp444
Kptn
D930028M14Rik
Alkbh6

Dkkl1
3110040N11Rik
Prcp
Neu3
Lrrc51
Trim6
Lyrm1
Sh2b1
Coro1a
AI467606
Oat
Pkd1l2
Chd9
Zfhx3
2810013P06Rik
Erich1
Plekha2
Brf2
Slc25a4
Abhd8
Calr3
Tom1
Fbxw9
Mt2
Ces2g
Atp6v0d1
Ranbp10
Cpne7
Ubash3b
Dmxl2
Plekho2
Abhd5
9230110C19Rik
Aplp2
Tbrg1
Oaf
Usp2
Gm5617
Rplp1
Rab11a
Rps27l
Tpm1
Elovl5
5830418P13Rik
Acad11
Cish
Rassf1
Hyal1
Tcta
Slc25a20

Shisa5
1700048O20Rik
Tsc22d3
Sat1
Pls3
Eda2r
Armcx2
Vezt
Cradd
Hint3
Marcks
Ccdc138
Ddit4
Dnajc12
Reep3
Mif
Itgb2
Lrrc3
Gpx4
Thop1
Ddit3
Esyt1
Cd63
Limk2
Ddc
Fstl4
Aldh3a2
Stat3
Dusp3
Zswim7
Zfp286
Med11
Camta2
Slc25a11
Nme2
Rsad1
Prr15l
Cwc25
Jup
Hdac5
Itga2b
Grn
Mapt
Fbf1
Ube2o
Afmid
Cbx8
Baiap2
Fam195b
Sirt7

Slc16a3
Foxn3
Ak7
Pacs2
Ptprn2
Arl4a
Ap4s1
Fut8
Adck1
Wdr35
Lpin1
Pqlc3
Hpcal1
Hbp1
Atxn7l1
3110056K07Rik
4930447C04Rik
Hspa2
Ltbp2
Eif2b2
Mlh3
Nudt14
Mta1
Dip2c
Dapk1
Atg10
Nqo2
Serpinb6a
Cast
Scamp1
Pik3r1
Klf6
Ggps1
Zfp184
Btn1a1
Hist1h1c
Acot13
Eci2
Zfp935
Zfp429
Arrdc3
Hexb
Vcl
Prkcd
Mettl6
Hacl1
Anxa8
Dydc2
Ero1l
Psme2

Slc39a14
Gm4285
Dap
Tg
Zfp385a
Derl1
Parp10
Vps28
Syngr1
Tef
Arsa
Aaas
Pfdn5
Prr13
Bdh1
Etv5
Dcbld2
Clic6
Sdf2l1
Hic2
Abcc5
Ndufb4
Cox17
Gm10789
Pigp
Tulp4
Agpat4
Zfand3
Serac1
Gtf2h5
Gm16702
Rab40c
Dusp1
Bak1
BC004004
Neu1
Mad2l1bp
Alkbh7
Yipf4
Sil1
Crem
Egr1
Sra1
Ppic
Adrb2
Acaa2
Snx32
Tmem2
Ablim1
Sipa1

Sac3d1
Gpr137
Bad
Cox8a
Ubxn1
Ostf1
Hhex
Pgam1
Got1
Sfxn3
Sfxn4
