# Supplementary material for: The histone methyltransferase Setd8 alters the chromatin landscape and regulates the expression of key transcription factors during erythroid differentiation
Source: Epigenetics Chromatin. 2020 Mar 16;13:16. doi: 10.1186/s13072-020-00337-9 (PMC7075014; doi:10.1186/s13072-020-00337-9)
Supplement: Supplementary file 2 — Additional file 2: Table S2. Gata2 target genes that are upregulated in setd8-mutant and have open chromatin higher in setd8-mutant. [file 13072_2020_337_MOESM2_ESM.pdf]

Gata2 target genes that are upregulated in setd8-mutant and have open chromatin higher in setd8-mutant

BTG2	FIS1
DDC	RPS9
CHD9	EGFL7
PIK3R1	ACOT7
ABHD5	JUP
HHEX	ELOVL5
FUT8	DAPK1
CREB3L1	MAD2L1BP
LMNA	PAFAH2
ARHGDIB	RAB11A
GPR137	GPN3
MFSD1	KLF6
METTL6	ADCK1
VAV3	TMBIM1
ARSA	ST7
	DDIT4
	SFXN3
	DYDC2
