# Supplementary material for: The histone methyltransferase Setd8 alters the chromatin landscape and regulates the expression of key transcription factors during erythroid differentiation
Source: Epigenetics Chromatin. 2020 Mar 16;13:16. doi: 10.1186/s13072-020-00337-9 (PMC7075014; doi:10.1186/s13072-020-00337-9)
Supplement: Supplementary file 3 — Additional file 3. Transcription factors predicted to regulate genes that contain a region of differential chromatin accessibility. [file 13072_2020_337_MOESM3_ESM.pdf]

Index	Name	P-value	Adjusted p-value	Z-score	Combined score
1	NFYA ENCODE	2.035e-7	0.00002055	-1.74	18.76
2	NFYB ENCODE	0.000001204	0.00006080	-1.66	16.09
3	GATA2 CHEA	0.000004516	0.0001520	-1.67	14.70
4	CEBPD ENCODE	0.00001127	0.0002277	-1.59	13.34
5	CTCF ENCODE	0.000008621	0.0002177	-1.53	12.89
6	TP53 CHEA	0.00002196	0.0003697	-1.49	11.81
7	CREB1 CHEA	0.00006146	0.0008868	-1.39	9.79
8	SP2 ENCODE	0.0001305	0.001464	-1.46	9.53
9	ZMIZ1 ENCODE	0.0001287	0.001464	-1.42	9.30
10	NANOG CHEA	0.0002236	0.002258	-1.38	8.38
11	STAT3 ENCODE	0.0003289	0.002768	-1.39	8.21
12	CEBPB ENCODE	0.0002927	0.002687	-1.37	8.08
13	PBX3 ENCODE	0.0004303	0.003343	-1.31	7.48
14	EGR1 CHEA	0.0006471	0.004668	-1.24	6.64
15	SP1 ENCODE	0.001134	0.006983	-1.21	5.99
16	GATA1 CHEA	0.0007766	0.005229	-1.14	5.98
17	NELFE ENCODE	0.001175	0.006983	-1.20	5.96
18	USF2 ENCODE	0.001430	0.007239	-1.11	5.47
19	TCF3 CHEA	0.001407	0.007239	-1.05	5.19
20	STAT3 CHEA	0.001453	0.007239	-0.97	4.77
21	SMC3 ENCODE	0.001505	0.007239	-0.96	4.73
22	NFE2L2 CHEA	0.001807	0.008295	-0.88	4.24
23	RUNX1 CHEA	0.002156	0.009466	-0.87	4.05
24	ZBTB7A ENCODE	0.002760	0.01162	-0.83	3.68
25	STAT5A ENCODE	0.003524	0.01424	-0.87	3.68
