# Supplementary material for: The histone methyltransferase Setd8 alters the chromatin landscape and regulates the expression of key transcription factors during erythroid differentiation
Source: Epigenetics Chromatin. 2020 Mar 16;13:16. doi: 10.1186/s13072-020-00337-9 (PMC7075014; doi:10.1186/s13072-020-00337-9)
Supplement: Supplementary file 4 — Additional file 4. Additional figures. [file 13072_2020_337_MOESM4_ESM.docx]

**Additional file 1: Figures:**


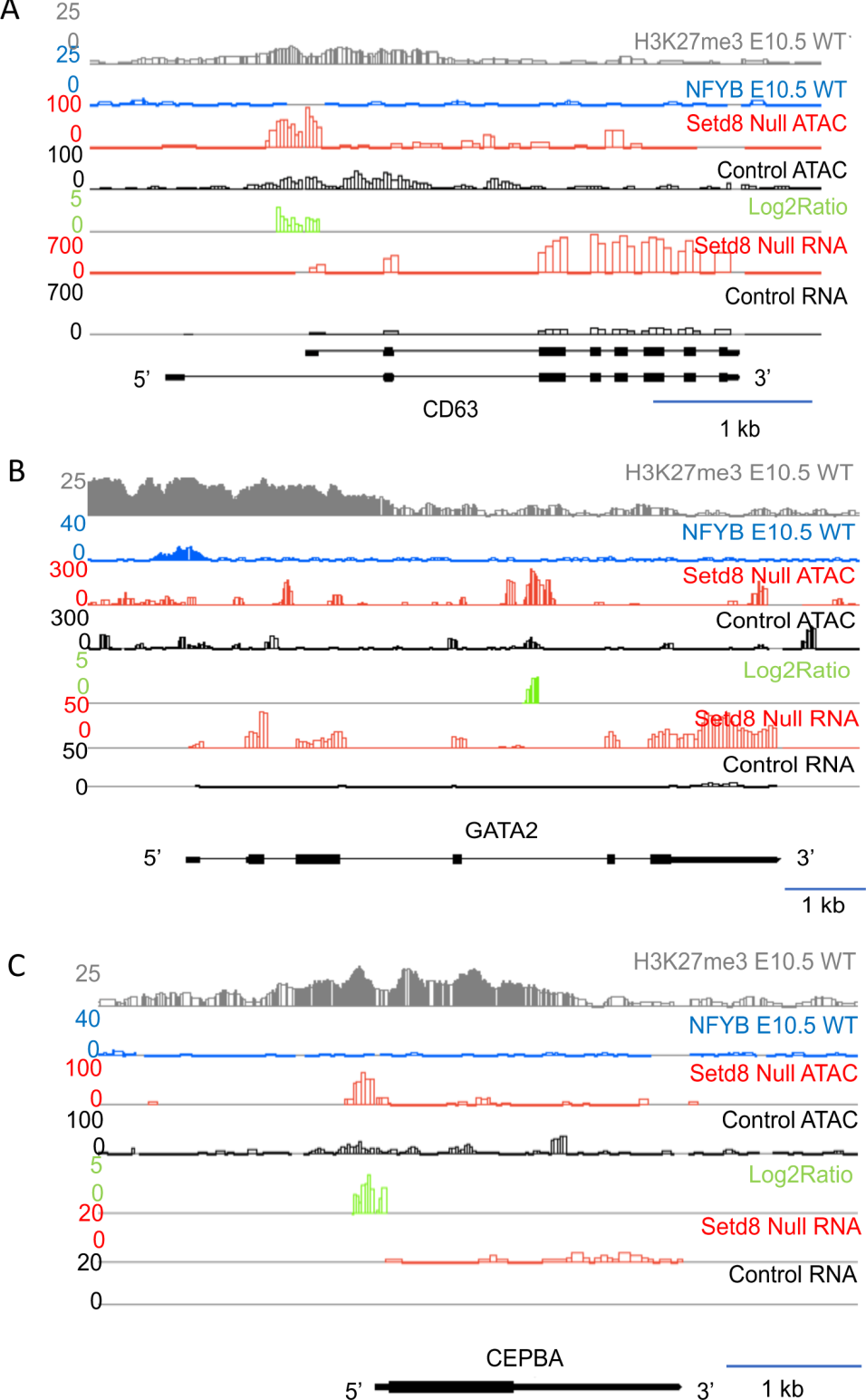


**Figure S1.** Chromatin landscape and mRNA expression at the (A) Cd63, (B) Gata2, and (C) Cepba loci.

**
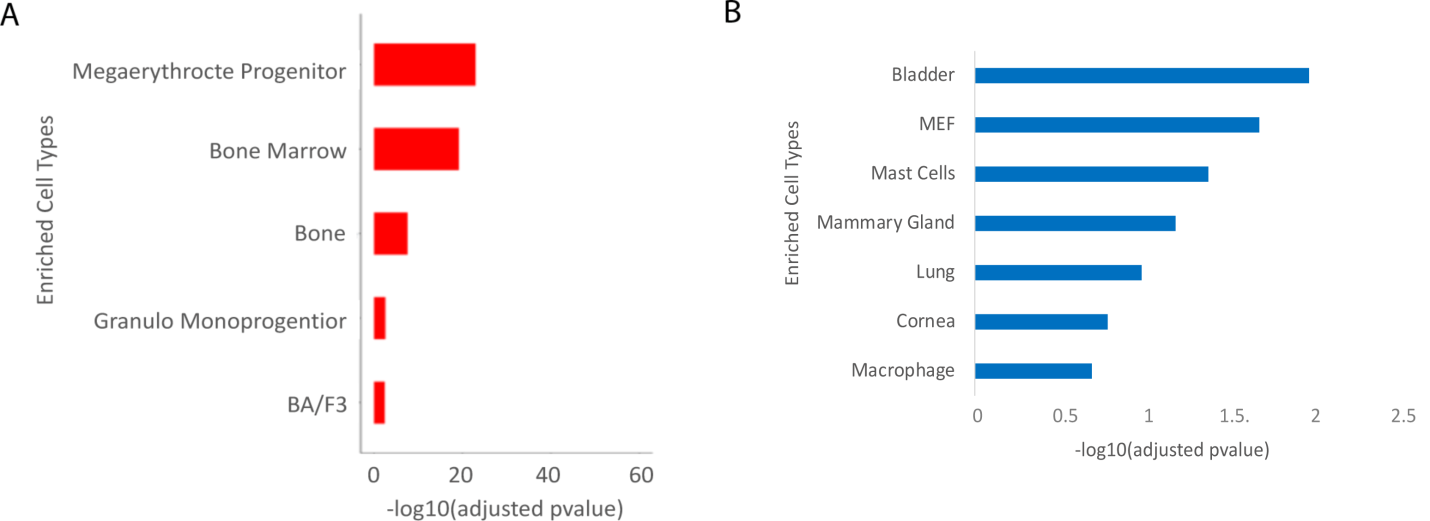
**

**Figure S2.** Cell-type enrichment analyses in *Setd8* mutant and control samples from Enrichr.

**
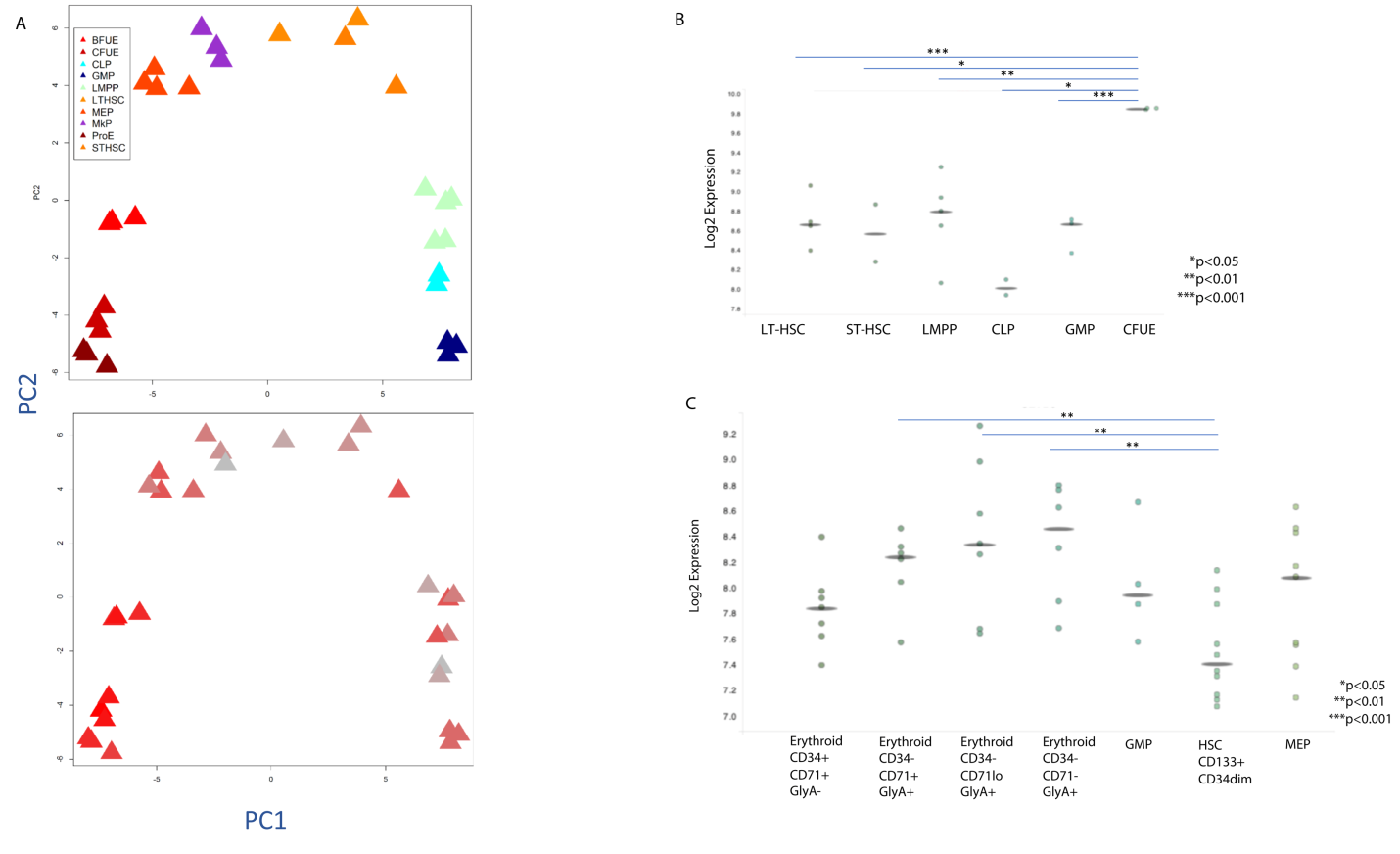
**

**Figure S3.** *Setd8* mRNA expression during murine and human erythropoiesis. (A) Upper panel: principle component analyses (PCA) of mRNA expression from GSE14833. Legend denotes the cell type of each sample. Lower panel: *Setd8* expression in each of the above populations, with darker red indicating higher Setd8 expression. (B) Expression of *Setd8* during murine hematopoiesis. Figure adopted from bloodspot.eu, using datasets [GSE14833](http://www.ncbi.nlm.nih.gov/geo/query/acc.cgi?acc=GSE14833) and [GSE6506](http://www.ncbi.nlm.nih.gov/geo/query/acc.cgi?acc=GSE6506). LT-HSC; Long term-hematopoietic stem cell, ST-HSC; short-term hematopoietic stem cell, LMPP; Lymphoid primed multipotent progenitors, CLP; common lymphoid progenitor, GMP; granulocyte monocyte progenitor, CFUE; colony forming unit erythroid. (C) Expression of *SETD8* during human erythropoiesis. Figure adopted from bloodspot.eu, using the [GSE24759](http://www.ncbi.nlm.nih.gov/geo/query/acc.cgi?acc=GSE24759) dataset. MEP; megakaryocyte erythroid progenitor, GMP; granulocyte monocyte progenitor, HSC; hematopoietic stem cell


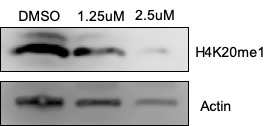


**Figure S4**. Western blot of H4K20me1 levels in CD34+ HSPCs treated with either DMSO or UNC0379 at indicated concentrations. Actin was used as a loading control.


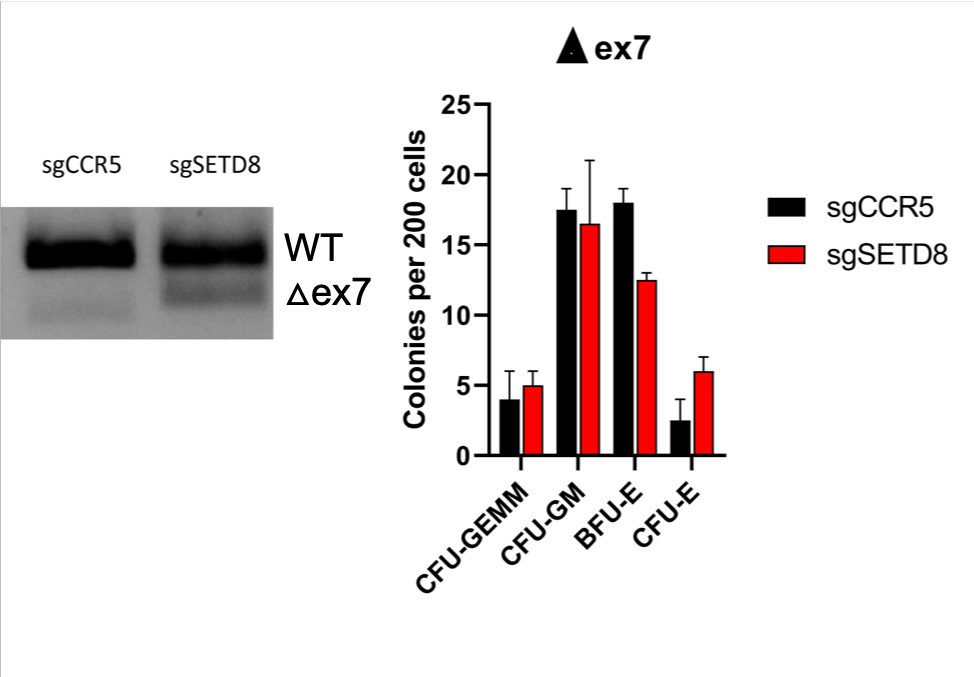


**Figure S5.** Colony forming ability following deletion of the methyltransferase domain of Setd8 (exon 7) via genome editing. Left figure shows PCR of the CCR5 locus and *SETD8* +4.4 exon 7 following genome editing via electroporation of Cas9-gRNA complexes, with wild type and genome edited bands as indicated. N=2 technical replicates.

**
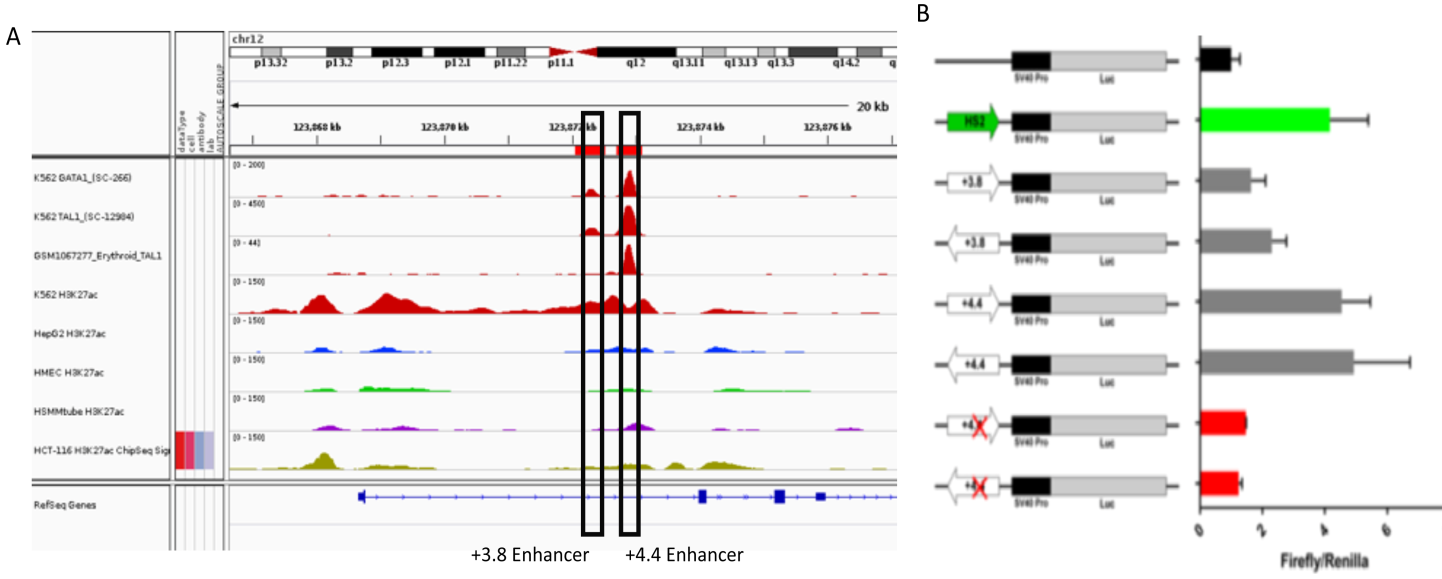
**

**Figure S6.** Identification of an enhancer that drives erythroid expression of *SETD8*. (A) Chromatin landscape of the human *SETD8* locus. The TAL1 track is from [GSE43625](https://www.ncbi.nlm.nih.gov/gds/?term=GSE43625%5BAccession%5D). K562, HepG2, HMEC, HSMMtube, and HCT116 tracks are from ENCODE. (B) Luciferase assays demonstrating the +4.4 enhancer has activity in both the forward and reverse ordination. Mutation of the GATA-TAL1 motif abrogates that activity. The HS2 enhancer from the b-globin locus is used as a positive control.

**
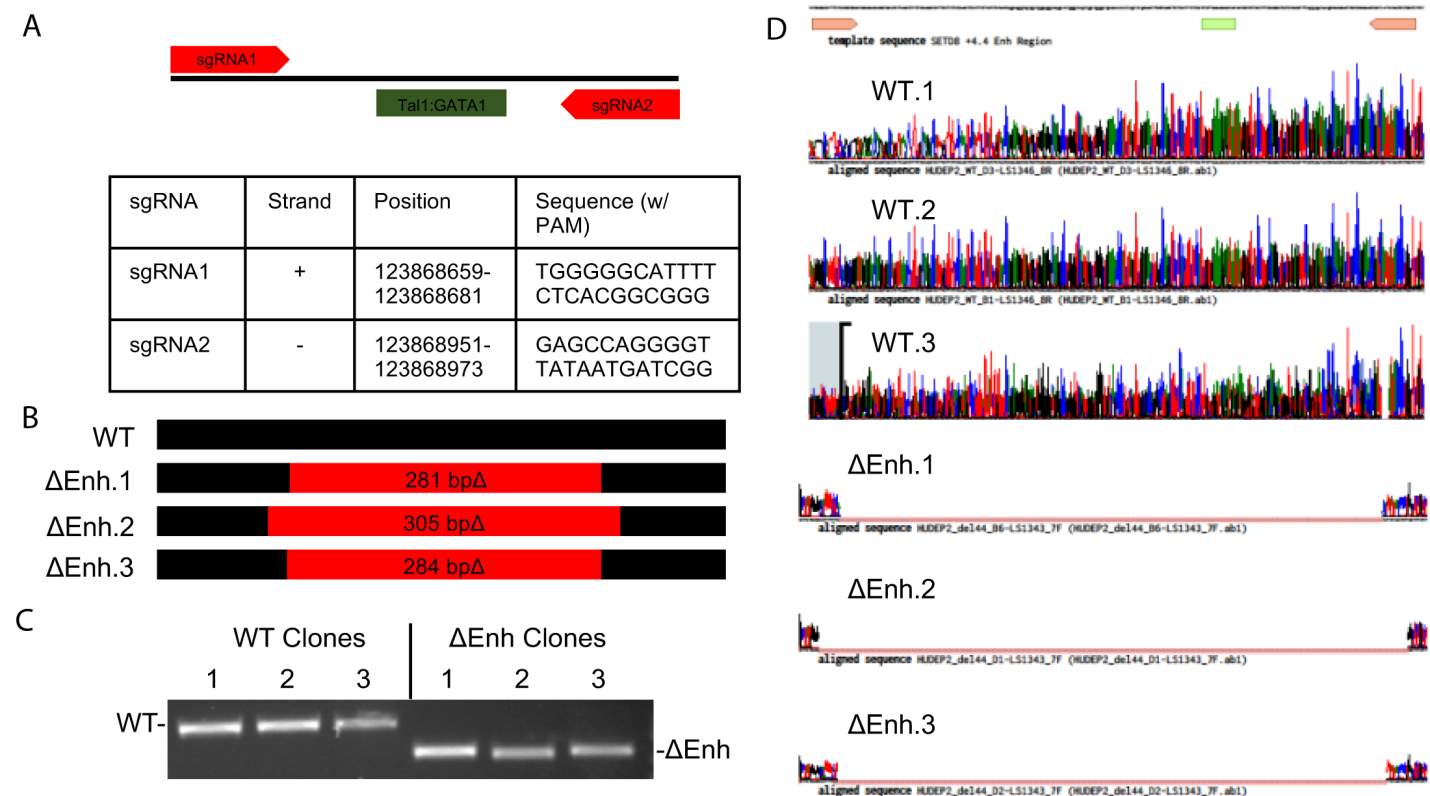
**

**Figure S7.** Deletion of the *SETD8* enhancer in HUDEP2 cells using genome editing. (A) Upper panel: location of gRNA relative to the GATA1-TAL1 motif at the center of the +4.4 enhancer. Lower panel: Sequence of the two gRNAs used to delete the *SETD8* erythroid enhancer. (B) Three clones with deletion of the *SETD8* enhancer were generated, with homozygous deletion of the enhancer as indicated. (C) PCR of the *SETD8* +4.4 erythroid enhancer, with smaller product confirming enhancer deletion in three distinct clones. (D) Sanger sequencing traces of the three wild-type control lines and the three enhancer deletion lines.

**
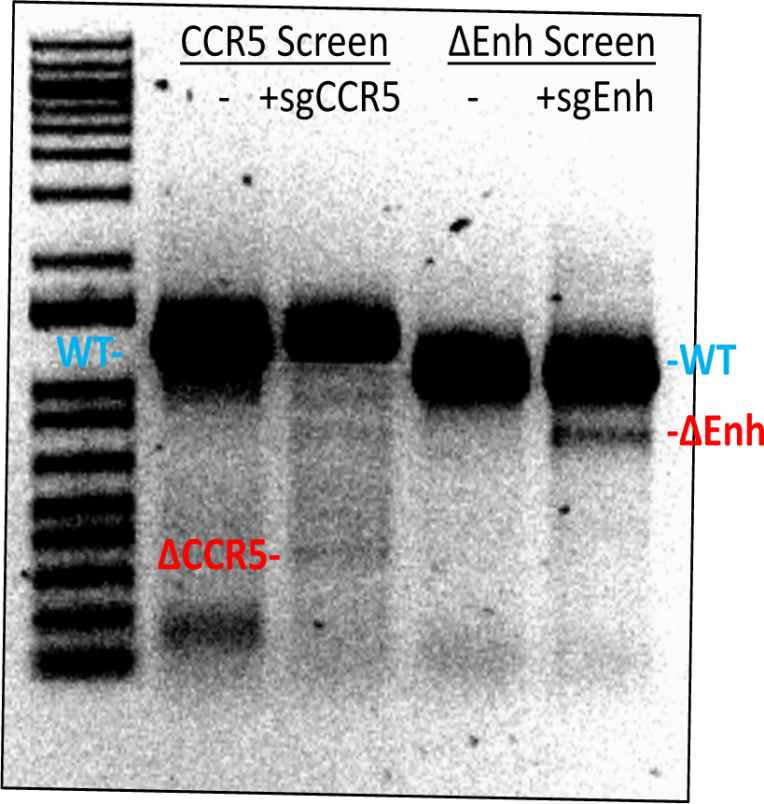
**

**Figure S8.** PCR of the CCR5 locus and the *SETD8* +4.4 enhancer following genome editing via electroporation of Cas9-gRNA complexes, with bands corresponding to wild type and genome edited regions as indicated.
